# Chronic use of psychotropic medications in breastfeeding women: Is it safe?

**DOI:** 10.1371/journal.pone.0197196

**Published:** 2018-05-21

**Authors:** Nirit Kronenfeld, Tomer ziv Baran, Maya Berlin, Nour Karra, Natalie Dinavitser, Rana Cohen, Yifat Wiener, Eyal Schwartzberg, Matitiahu Bercovitch

**Affiliations:** 1 Department of Pharmacy, The Hebrew University of Jerusalem, Jerusalem, Israel; 2 Department of Clinical Pharmacology Unit, Assaf Harofeh Medical Center, Zerifin, Affiliated to Sackler School of Medicine, Tel-Aviv University, Tel-Aviv, Israel; 3 Department of Epidemiology and Preventive Medicine, School of Public Health, Sackler Faculty of Medicine, Tel Aviv University, Tel Aviv, Israel; 4 Department of Obstetric and Gynecology, Assaf Harofeh Medical Center, Zerifin, Affiliated to Sackler School of Medicine, Tel-Aviv University, Tel-Aviv, Israel; 5 Department of Pharmacy, Ben Gurion University of the Negev, Beer-Sheba, Israel; TNO, NETHERLANDS

## Abstract

**Background:**

Current knowledge regarding chronic use of psychotropic medications during breastfeeding is limited. The objective of this study was to evaluate the long-term effects of psychotropic monotherapy use during lactation on the breastfed infant.

**Materials and methods:**

In this prospective study, we followed 280 infants whose mothers contacted the Drug Consultation Center (DCC) at Assaf Harofeh Medical Center between January 2011 and December 2015, seeking information regarding the chronic use of psychotropic medications during lactation. This group was compared with a group of 152 callers, who inquired evidence regarding the use of antibiotics compatible with breastfeeding. Information on adverse effects, physical measures and gross motor developmental milestone achievements of the breastfed infants was obtained during a follow-up telephone interview. At follow up, the median age of the infants in the Psychotropic-drug group was 20 (11–33) months versus 36 (20–48) months in the Antibiotic group (p < 0.001). The outcomes were compared between the groups followed by a propensity score matching to control for difference in baseline characteristics.

**Results:**

At follow-up, no significant differences between infants in the two groups were observed with regard to height, weight, head circumference and weight-length ratio percentile (p = 0.339, p = 0.223, p = 0.738, p = 0.926, respectively). Children in both groups were, according to their parents, within the normal developmental range for all milestones, according to the Denver Developmental Scale. Use of psychotropic medications during breastfeeding was not significantly associated with adverse reactions. After propensity score matching (n = 120 pairs) to control for differences in baseline characteristics and the length of lactation, only one significant difference was reported, sleepiness in infants in the study group (7/120) and none in the comparison group (p = 0.008).

**Conclusions:**

Chronic use of psychotropic monotherapy during lactation is associated with normal growth and gross motor developmental as by milestone achievements reported by parents. Sleepiness was reported, it seemed self-limited with no developmental effect.

## Introduction

Depression and other psychiatric disorders are common during adulthood, affecting women of childbearing ages. Most episodes (40%) arise during the postpartum period with 33.4% initiating during pregnancy and 26.5% of the events reported to antedate pregnancy [1]. Antidepressants are commonly used in the treatment of depression during and after pregnancy [2].

Women with a history of a psychiatric condition are at a higher risk of developing depression throughout pregnancy and postpartum [3]. Untreated psychiatric conditions during pregnancy and the postpartum period may have a deleterious effect on the mother and her child [4]. This may result in impaired bonding, lower intelligence quotients and behavioral problems [5, 6]. Therefore, it is often mandatory to continue therapy after delivery [7].

Exclusive breast-feeding for the first 6 months, followed by continued breast-feeding with the addition of complementary iron-enriched solid foods, is recommended by various organizations as the best nutrition until at least 12 months [8–10].

Uncertainty among medical professionals concerning the management of psychotropic therapy in nursing mothers can lead to suboptimal dosage prescription and poor maternal compliance [11]. Most psychotropic medications are secreted in breastmilk, however, little is known about the long-term effects of such medications during breastfeeding on the infant [10]. Data on the chronic use of these agents is limited to few case reports [12], selective drug(s) [13–17] or studies with small sample sizes (up to 30 breastfeeding women) [18–21].

The aim of the study is to prospectively follow infants whose mothers contacted the drug consultation center (DCC) to inquire about the safety of psychotropic medications during breastfeeding.

The DCC is run by a team of pharmacists and physicians who provide information and guidance to pregnant or lactating women and to health care professionals regarding risks of exposure of the fetus and or the breastfed infant to drugs, chemicals, and nutritional supplements. The DCC also provides information concerning adverse drug reactions, drug interactions and drug dosage. The information is provided by a telephone information service.

## Materials and methods

### Study cohort and design

A prospective cohort study was carried at the Drug Consultation Center (DCC) at Assaf Harofeh Medical Center, Israel. The DCC provides information and guidance to pregnant or lactating women and to health care professionals, via a telephone information service. The average number of general calls to the DCC from 2011 to 2015 was 8,221 calls per year, of which 5,183 (63%) involved direct inquiries concerning pregnancy and lactation.

We consecutively enrolled women who called the DCC between January 2011 and December 2015.

The chronically exposed group included postnatal women requesting information on psychotropic medications during breastfeeding. This group was compared with breastfeeding women treated with short-term (up to 10 days) antibiotic monotherapy, considered safe while breastfeeding [22, 23].

In this study, women were not discouraged or encouraged to take any specific medication, nor were they given any advice on less risky medications. The information was not based on the severity of the illness for which the antibiotic or psychotropic medication was prescribed, but on the specific medication.

### Data measures

Using a structured questionnaire, baseline information and information subsequent to treatment initiation was collected from the initial call. Data collected included maternal age, profession, psychotropic drug dosage, indication and duration of therapy, age and medical status of the infant. Information on baseline characteristics and covariates that may suggest abnormal development of the infant, including chronic maternal illness, smoking, alcohol and drug consumption habits, gestational age at birth, infant birth weight, and perinatal complications, were obtained.

A follow-up telephone call was performed within several months to five years after the first call. Using a structured questionnaire, detailed information regarding the extent of drug exposure during pregnancy and lactation, dosages, indications, duration of therapy and pattern of breastfeeding including exclusivity and degree of formula supplementation was obtained. Adverse reactions of the infants were recorded and were defined as changes in the infant's condition observed by the mother or physician during maternal drug therapy [24]. These included rash, restlessness, diarrhea, shivering, seizures, sleepiness, excessive crying, or need for consultation with a pediatrician. Information on weight gain, height, and head circumference was detailed by the parent from the well-baby clinic card. Height, weight, head circumference percentiles and weight- length ratio of the breastfed infants were evaluated according to growth curves of the Israeli Ministry of Health. Infant's progress with respect to meeting gross motor developmental milestone was evaluated in each case, using the Denver Developmental Scale, as previously reported by our group [25]. In the follow-up interview, the developmental assessment was detailed by the mother based on the "well-baby" clinic card.

Exclusion criteria included women who did not take psychotropic medication, women who decided not to breastfeed, or women who used polytherapy psychotropic medications or antibiotics. Patients were considered as lost to follow-up if they could not be reached after 5 phone call attempts at different daily hours.

### Sample size

We used α = 1% and power of 80% to calculate the sample size. In order to identify medium effect size (d = 0.5) in continuous variables, ninety- six patients in each group were needed.

In order to identify medium effect size (w = 0.3) in dichotomial variables, sixty- five patients in each group were needed [26].

### Statistical analysis

The statistical analysis was conducted using IBM SPSS Statistics for Windows, version 23 (IBM Corp., Armonk, N.Y., USA) and R version 3.2.3 (R Foundation for Statistical Computing, Vienna, Austria). All statistical tests were two tailed and p < 0.05 was considered as statistically significant. Continuous variables are presented as mean and standard deviation (SD) or median and interquartile range (IQR). Continuous variables were compared between groups using t test or Mann-Whitney test and categorical variables were compared using Chi square or Fisher’s exact test, as appropriate. Multivariate analysis was performed using logistic regression analysis to examine the association between medications and outcomes, with adjustment for other variables. Odds ratio (OR) and 95% confidence interval (CI) were reported. Variants that presented a significance of p < 0.2in the univariate analysis were included in the multivariate analysis. Variants that are known in the literature as potential confounders were also included in the multivariate analysis. Further analyses were performed after propensity score matching of the chronic psychotropic medication and the antibiotic groups. Propensity score was calculated using multivariate logistic regression as the probability of treatment with psychotropic medication [27]. The multivariate logistic regression model included age, number of children, education, profession, type of lactation, lactation weeks and lactation only weeks. The propensity score in our study is the probability that a child will be exposed to psychotropic medication according to maternal variables. An absolute 5% difference in the propensity score was considered as an acceptable threshold value for matching [27]. After matching, the chronic psychotropic medication group and antibiotic group were compared using Wilcoxon signed ranks test (continuous variables) and McNemar test categorical variables. The matching process was evaluated using standardized difference plot and mirrored histogram [27].

The study was approved by Assaf Harofeh Medical Center Ethics Committee before the research was started (The number of the protocol is: 0215–15). Informed consent was obtained from all individual participants included in the study.

## Results

### Study cohort

Six hundred twenty-six women contacted the DCC during the study period seeking information regarding psychotropic or antibiotic use during lactation. Five hundred forty-seven women gave their consent to participate in the study and 79 refused. Of the 547 women who agreed to participate in the study, 395 called to obtain data on chronic use of psychotropic medications and 152 were seeking information on antibiotic use during breastfeeding (S1 Fig).

The overall response rate was 547 out of 626 (87.4%). Three hundred and ninety- five women out of 466 (84.7%) in the chronic psychotropic medication group and 152 out of 160 (95%) in the antibiotic group have responded (S1 Fig).

No significant differences were observed between women who gave their consent to participate and those who declined (S1 Table). One hundred and fifteen women were excluded from the chronic psychotropic medication arm (S1 Fig). The final study included 432 breastfeeding women. The psychotropic medication group included 280 breastfeeding women who were compared to 152 women in the antibiotic medication group. Women in the Psychotropic group were contacted within a median of 32 (range, 16–42) months following the initial call, and those in the Antibiotic group were contacted at a median of 35 (16–48) months following the first call (p = 0.09). The median age of the infants in the Psychotropic group at follow up was 20 (11–33) months versus 36 (20–48) months in the Antibiotic group (p < 0.001).

#### Baseline characteristics

The most common diagnoses for which treatment was offered in the Psychotropic group were depression (59.6%) and anxiety (33.9%). There were 193 (68.9%) women who were exposed to selective serotonin reuptake inhibitors (SSRIs), 37 (13.2%) to benzodiazepines, 23 (8.2%) to serotonin–norepinephrine reuptake inhibitors, 11 (3.9%) to tricyclic antidepressant, 6 (2.1%) to first generation antipsychotics, 5 (1.8%) to second generation antipsychotics, 2 (0.7%) to other antipsychotics, and 2 (0.7%) to norepinephrine reuptake inhibitors. The dosage of psychotropic medications was within the recommended dose for the specific indication.

In the Antibiotic group, 58 (38.2%) breastfeeding women were exposed to metronidazole, 48 (31.6%)—new macrolides, 22 (14.5%)- quinolones, 12 (7.9%)—nitrofurantoin, 7 (4.6%)—fosfomycin, 4 (2.6%)—doxycycline, 1 (0.7%)—clindamycin, and 1 (0.7%) to cefuroxime.

Women in the Psychotropic group had a higher educational background (p<0.001), less children (p = 0.003) and their profession varied from those in the Antibiotic group (p = 0.002). No other significant differences were observed between women in both groups (Table 1).

**Table 1:** Baseline characteristics of mothers and infants exposed to psychotropic medications or antibiotics during breastfeeding before and after matching.

	Before matching			After matching		
Parameter	Psychotropic group (N = 280)	Antibiotic group (N = 152)	P value	Psychotropic group (N = 120)	Antibiotic group (N = 120)	P value
Maternal age, median (IQR), year	35 (32–39)	35 (31–38)	0.14	34 (31–38)	34 (31–38)	0.93
Chronic maternal illness, No. (%)						
Hypothyroidism	13 (4.6%)	3 (2%)	0.16	3 (2.5%)	3 (2.5%)	>0.99
Hematology	5 (1.8%)	4 (2.6%)	0.73	2 (1.7%)	4 (3.3%)	0.68
Gastrointestinal	3 (1.1%)	4 (2.6%)	0.25	3 (2.5%)	2 (1.7%)	>0.99
Respiratory	4 (1.4%)	0 (0%)	0.30	4 (3.3%)	0 (0%)	NA
Cardiovascular	2 (0.7%)	0 (0%)	0.54	1 (0.8%)	0 (0%)	NA
Diabetes	3 (1.1%)	1 (0.7%)	>0.99	2 (1.7%)	1 (0.8%)	>0.99
Dermatology	2 (0.7%)	0 (0%)	0.54	1 (0.8%)	0 (0%)	NA
Education ≤ 12 years, No. (%)	99 (35.4%)	29 (19.1%)	<0.001	28 (23.3%)	28 (23.3%)	>0.99
Maternal Profession, No. (%)			0.002			0.82
House hold	67 (23.9%)	13 (8.6%)		14 (11.7%)	12 (10%)	
Office work	126 (45%)	73 (48%)		52 (43.3%)	62 (51.7%)	
Education	51 (18.2%)	35 (23%)		31 (25.8%)	26 (21.7%)	
Psychology / Social work	10 (3.6%)	7 (4.6%)		4 (3.3%)	6 (5.0%)	
Medical staff (doctors, nurses, etc.)	26 (9.3%)	24 (15.8%)		19 (15.8%)	14 (11.7%)	
Alcohol consumption during pregnancy, No. (%)	2 (0.7%)	1 (0.7%)	>0.99	2 (1.7%)	1 (0.8%)	>0.99
Smoking cigarette during pregnancy, No. (%)	8 (2.9%)	0 (0%)	0.05	6 (5%)	0 (0%)	NA
No. of children, median (IQR)	2 (1–3)	2 (2–3)	0.003	2 (1–3)	2 (2–3)	0.63
Gender of the child, No. (%)			0.38			>0.99
Male	136 (48.6%)	80 (53%)		66 (55%)	65 (54.2%)	
Female	144 (51.5%)	71 (47%)		54 (45%)	55 (45.8%)	
Age of the child at follow up, median (IQR), month	20 (11–33)	36 (20–48)	<0.001	18 (10–32)	33 (18–48)	<0.001
Type of lactation, No. (%)			<0.001			>0.99
Exclusive lactation	98 (35%)	93 (61.2%)		66 (55%)	67 (55.8%)	
Partial lactation	182 (65%)	59 (38.8%)		54 (45%)	53 (44.2%)	
Duration of lactation, median (IQR), week						
Lactation (general)	24 (12–40)	36 (24–59)	<0.001	28 (20–48)	32 (20–48)	0.36
Exclusive lactation	12 (1–24)	24 (16–24)	<0.001	24 (2.5–24)	24 (8–24)	0.38

### Comparison of pregnancy and perinatal complications

There were no statistical differences in the overall rate of maternal pregnancy complications between women treated with psychotropic drugs and those receiving antibiotics (p = 0.661).

The rate of infants’ complications during the neonatal period was higher in the Psychotropic group: 19 (6.78%) compared to one case (0.65%) in the control group, (p = 0.003). Fetal distress was higher in infants belonging to the Psychotropic group: 15 (5.35%) versus 0 (0%) in the comparison group (p = 0.002) (S2 Table).

### Comparison of outcome measures

No significant difference was observed between infants in the two groups regarding percentile of height, weight, head circumference and weight- length ratio of the children in each arm (S3 Table).

Fourteen infants in the Psychotropic group (5%) experienced adverse reactions versus 7 infants (4.6%) in the Antibiotic group (p = 0.855) (Table 2). A significant difference was only observed for diarrhea; 0 versus 7 (4.6%), (p = 0.001), for the Psychotropic and Antibiotic groups, respectively. Eight infants in the Psychotropic group were sleepy as compared to none in the Antibiotic group. However, the difference was not statistically significant (p = 0.055). In all 8 cases of a sleepy child, the mothers were treated with psychotropic medication throughout pregnancy. Neonatal sleepiness was observed shortly after birth in 6 of the cases, as reported by the mothers (escitalopram- 2 cases, paroxetine, amitriptyline, lorazepam, and sertraline, 1 case each). One case (with escitalopram) was noted at the age of 3 days and another case (with citalopram) was described 4 days after birth. In all cases, sleepiness resolved spontaneously within 24 hours, with no developmental effect.

**Table 2:** Frequency of adverse reactions the infant experienced while being exposed during breastfeeding to one of the psychotropic medications or antibiotics before and after matching.

	Before matching			After matching		
Type of adverse reaction	Psychotropic group (N = 280)	Antibiotic group (N = 152)	P value	Psychotropic group (N = 120)	Antibiotic group (N = 120)	P value
Any Adverse reaction	14 (5%)	7 (4.6%)	0.85	9 (7.5%)	6 (5.0%)	0.61
Sleepiness	8 (2.9%)	0 (0%)	0.05	7 (5.8%)	0 (0%)	0.008
Poor weight gain	4 (1.4%)	0 (0%)	0.30	2 (1.7%)	0 (0%)	0.16
Shivering	2 (0.7%)	0 (0%)	0.54	0 (0%)	0 (0%)	NA
Seizures	0 (0%)	0 (%)	NA	0 (0%)	0 (0%)	NA
Excessive crying	0 (0%)	0 (%)	NA	0 (0%)	0 (0%)	NA
Rash	0 (0%)	0 (%)	NA	0 (0%)	0 (0%)	NA
Diarrhea	0 (0%)	7 (4.6%)	0.001	0 (0%)	6 (5.0%)	0.01

Data are expressed as No. (%)

Abbreviations: NA = not applicable

In multivariate analysis, no association was found between exposure to psychotropic medications during breastfeeding and the risk of adverse reactions to the children compared to the antibiotics-treated group (Odds ratio = 1.098, 95% confidence interval 0.432–2.792, p = 0.844).

Statistically significant differences were observed between the groups regarding some milestones. However, children in both groups were, according to their parents, within the normal developmental range for all milestones (Table 3).

**Table 3:** Comparison between the two groups according to age (months) at which each milestone was acquired before and after matching.

	Before matching			After matching		
Milestone	Psychotropic group (N = 280)	Antibiotic group (N = 152)	P value	Psychotropic group (N = 120)	Antibiotic group (N = 120)	P value
Smile	1.55, 2 (1–2)	1.32, 1 (1–2)	<0.001	1.55, 2 (1–2)	1.32, 1 (1–2)	<0.001
Lift head	2.74, 3 (3–3)	2.56, 3 (2–3)	0.001	2.74, 3 (3–3)	2.57, 3 (2–3)	0.04
Sitting	7.51, 8 (6–9)	7.93, 8 (7–9)	0.01	7.49, 7 (6–9)	7.92, 8 (7–9)	0.03
Crawling	7.48, 8 (7–8)	7.17, 7 (6–8)	0.02	7.53, 8 (7–8)	7.15, 7 (6–8)	0.03
Standing	10.2, 10 (9–11)	10.03, 10 (9–11)	0.06	10.03, 10 (9–11)	10.08, 10 (9–11)	0.74
First word	11.1, 11 (10–12)	11.34, 11 (10–12)	0.19	11.08, 11 (10–12)	11.44, 11.5 (10–12)	0.13
Walking	12.79, 13 (12–14)	12.53, 12 (11.5–13)	0.12	12.73, 13 (12–14)	12.55, 12 (11–13)	0.64

Data are expressed as mean, median (IQR)

### Comparison of outcome measures after propensity score matching

In the Psychotropic-drug group, women breastfed for a shorter duration (median 24 weeks), as compared to the Antibiotic group (median 36 weeks), (p<0.001). Ninety-eight (35%) women in the Psychotropic group were on exclusive lactation as compared to 93(61%) in the Antibiotic group (p<0.001) (Table 1). Therefore, in addition to the multivariate analysis noted above, propensity score matching was utilized to reduce or eliminate the effects of confounding in our observational cohorts.

The baseline characteristics of the two propensity matched groups (120 pairs) are presented in Table 1. The evaluation of the matching process is described in S2 Fig and S3 Fig. The standardized differences of mother’s respiratory illness and cigarette smoking during pregnancy were over 0.2. However, these baseline characteristics were rare. Four women (3.3%) in the Psychotropic group experienced respiratory illness versus 0 women in the Antibiotic group. Six women (5%) in the Psychotropic group smoked cigarettes during pregnancy, versus none in the Antibiotic group.

The results after propensity score matching between the groups were similar to the results from the total cohort. Growth percentiles were not significantly different between the propensity score-matched groups (S3 Table). The rates of any adverse reaction were not significantly different between the matched groups. However, when specific adverse effects were analyzed separately, sleepiness was reported in 7 (5.8%) infants in the Psychotropic group and none in the Antibiotic group (p = 0.008); 3 infants were exposed during breastfeeding to escitalopram, 2 to paroxetine, and 1 to citalopram and lorazepam, each. The dosage of these medications was within the recommended dose for the specific indication. Diarrhea was not reported in the Psychotropic group, as compared to 6 infants (5%) in the Antibiotic group (p = 0.014) (Table 2). Gross developmental milestones were within the normal range in both groups (Table 3).

### Low milk production

At follow-up telephone call, mothers in both groups were asked about milk production. Low milk production was reported by 13 (4.6%) women in the psychotropic group versus 5 (3.3%) in the antibiotics group (p = 0.501).

## Discussion

Using a multivariate analysis, no association was observed between exposure of children to psychotropic medications during breastfeeding and the risk of adverse reactions compared to children exposed to antibiotics during breast feeding (OR = 1.098, 95% CI 0.432–2.792, p = 0.844). The results after propensity score matching between the groups were similar to the results from the total cohort. The rates of any adverse reaction in the infants were not significantly different between the matched groups. When analyzed separately for specific adverse effects, sleepiness was reported in 7 (5.8%) infants in the Psychotropic group and none were encountered in the Antibiotic group (p = 0.008). In all 7 cases, the mothers were treated with psychotropic medication throughout pregnancy. However, there are many reasons why babies are sleepy in the first few days of life, and this may not be a definite adverse event. Since sleepiness was observed shortly after birth in most cases and resolved spontaneously within 24 hours, it is possible that this symptom was part of the well-known phenomena of poor neonatal adaptation described previously with the use of psychotropic medications throughout pregnancy, where symptoms develop within 48 hours after birth and continue for 2–6 days [28–31].

Diarrhea was not reported in the Psychotropic group, as compared to 6 infants (5%) in the Antibiotic group (p = 0.014) (Table 2). Our results are consistent with previous studies and case reports reporting no or insignificant adverse reactions in the nursing infant while being exposed to psychotropic medications. However, these studies and case reports included relatively small number of patients [12–18, 19–23, 32].

The median age of the infants in the Psychotropic group at follow up was 20 (11–33) months versus 36 (20–48) months in the Antibiotic group (p < 0.001). Although statistically significant differences were observed between the groups for some milestones (Table 3), children in both groups were within the normal developmental range for all milestones, a finding previously reported with a limited number of infants [21, 33]. There is no published data in the literature addressing the association between chronic use of psychotropic medications during breastfeeding and height, weight, head circumference and weight-length ratio percentile in nursing infants. Comparative analysis of the two groups in this study, did not find significant differences in the various growth measures. It is unlikely therefore, that psychotropic therapy during breastfeeding will result in growth retardation in the nursing infant.

Women from the Psychotropic group breastfed for a lesser extent of time (median 24 weeks), compared to the Antibiotic group (median 36 weeks), (p<0.001). Ninety-eight (35%) women in the Psychotropic group chose exclusive lactation as compared to 61% in the Antibiotic group (p<0.001). After propensity score matching, the baseline characteristics of the mothers and infants were more comparable, especially for the type and duration of breastfeeding (S2 Fig). The results after propensity score matching between the groups (120 pairs) were similar to the results of the total cohort. Growth percentiles were not significantly different between the propensity score-matched groups and gross developmental milestones achieved by children in both groups and were within the normal range. Infants chronically exposed to psychotropic monotherapy during breastfeeding were more likely to be somnolent than unexposed ones, providing reassurance that exposure did not affect infant growth or gross motor development.

The overall rate of maternal complications was not different between the groups, but the rate of some infants' complications during the neonatal period was higher in the Psychotropic group. Use of antidepressants, such as SSRI's, tricyclic antidepressant and venlafaxine during pregnancy, may increase the risk of respiratory distress syndrome, hypoglycemia, low birth weight and seizures in the infant [34]. However, in recent years, it has become increasingly evident that an untreated, underlying psychiatric disorder by itself increases the risk for a negative pregnancy outcome.

The rates of low milk production by the mother were not significantly different between the groups, but numerically were larger in the psychotropic group (13 women versus 5 in the antibiotic group). Indeed, there is some evidence that SSRIs may interfere with lactogenesis.

The strength of this study is in its large prospective cohort design, where reliable information regarding several classes of psychotropic drugs and their effect during lactation on the infant was collected.

Detailed initial and follow up questionnaires were filled out, where potential confounders were also adjusted for in the subsequent multivariate analysis.

The study has its limitations. The data was collected using a structured questionnaire during telephone interviews. Given that the information came directly from the mother, the data is potentially affected by an information bias. However, infantile weight, height, and head circumference was detailed by the parent from the well-baby clinic card and would not be subject to recall bias.

Evaluating the gross motor development of the infants based on telephone screening, might be unreliable in predicting less severe or specific developmental problems nor will it identify subtle developmental differences. Drug concentrations in the mother's milk or in infant serum were not measured, since it was not feasible to perform such measurements.

## Conclusion

Based upon telephone evaluation, the use of psychotropic medications during lactation is associated with normal growth and gross motor developmental as by milestone achievements reported by parents. Sleepiness was reported, however, it seemed self-limited with no developmental effect. Larger studies and more objective data is needed to confirm these findings.

## Supporting information

S1 Fig Distribution of women in the study. (DOC)

S2 Fig Standardized differences plot, baseline Characteristics of women exposed to psychotropic medications or antibiotics during breastfeeding before and after matching. Circle indicate before; triangles indicate after.
(DOCX)

S3 Fig Mirrored histogram of distribution of propensity scores for Psychotropic and Antibiotic groups. (DOCX)

S1 Table Baseline Characteristics of Mothers included in the Psychotropic group and Antibiotic group who responded and non-responded to the follow up interview. (DOCX)

S2 Table Rate of perinatal complications in women exposed to psychotropic medications or antibiotics during childbirth. (DOCX)

S3 Table Height, weight, head circumference and weight- length ratio percentile at follow up of the breastfed infant before and after matching. (DOCX)

S1 File Baseline information and information subsequent to treatment initiation of women and their infants in the psychotropic and the antibiotic groups. (XLSX)

S2 File Follow up Questionnaire. (DOC)
