# Correction: Chronic use of psychotropic medications in breastfeeding women: Is it safe?

**DOI:** 10.1371/journal.pone.0199906

**Published:** 2018-06-25

**Authors:** Nirit Kronenfeld, Tomer ziv Baran, Maya Berlin, Nour Karra, Natalie Dinavitser, Rana Cohen, Yifat Wiener, Eyal Schwartzberg, Matitiahu Berkovitch

The ninth author’s name is spelled incorrectly. The correct name is: Matitiahu Berkovitch.
