# Supplementary figures and images for: Chronic use of psychotropic medications in breastfeeding women: Is it safe?
Source: PLoS One. 2018 May 21;13(5):e0197196. doi: 10.1371/journal.pone.0197196 (PMC5962050; doi:10.1371/journal.pone.0197196)

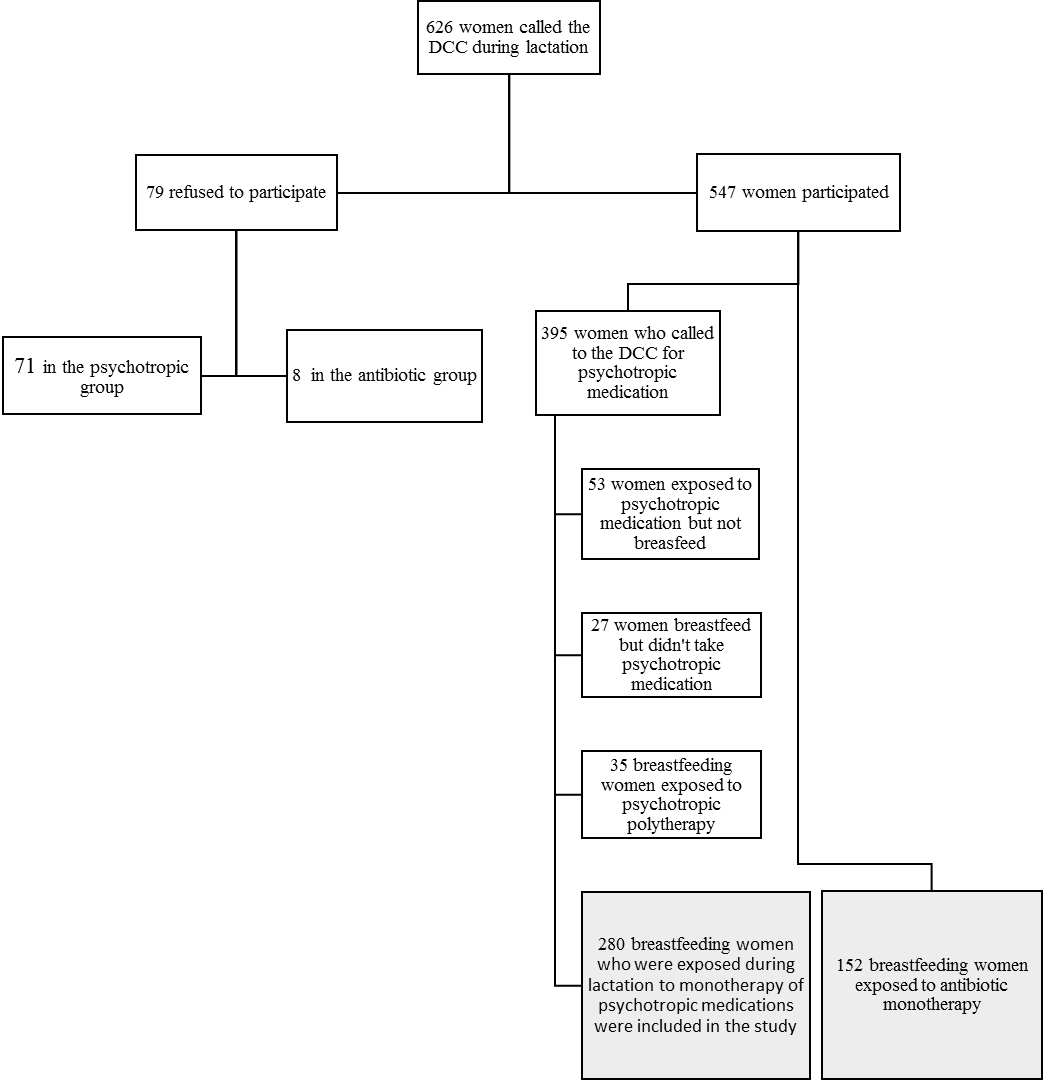
**Figure 1.** Distribution of women in the study.

Supplement: S1 Fig — (DOC) [file pone.0197196.s001.doc]
