# Supplementary material for: Chronic use of psychotropic medications in breastfeeding women: Is it safe?
Source: PLoS One. 2018 May 21;13(5):e0197196. doi: 10.1371/journal.pone.0197196 (PMC5962050; doi:10.1371/journal.pone.0197196)
Supplement: S2 Fig — Circle indicate before; triangles indicate after. (DOCX) [file pone.0197196.s002.docx]

**S2 Fig.** Standardized differences plot, baseline Characteristics of women exposed to psychotropic medications or antibiotics during breastfeeding before and after matching. Circle indicate before; triangles indicate after.

**
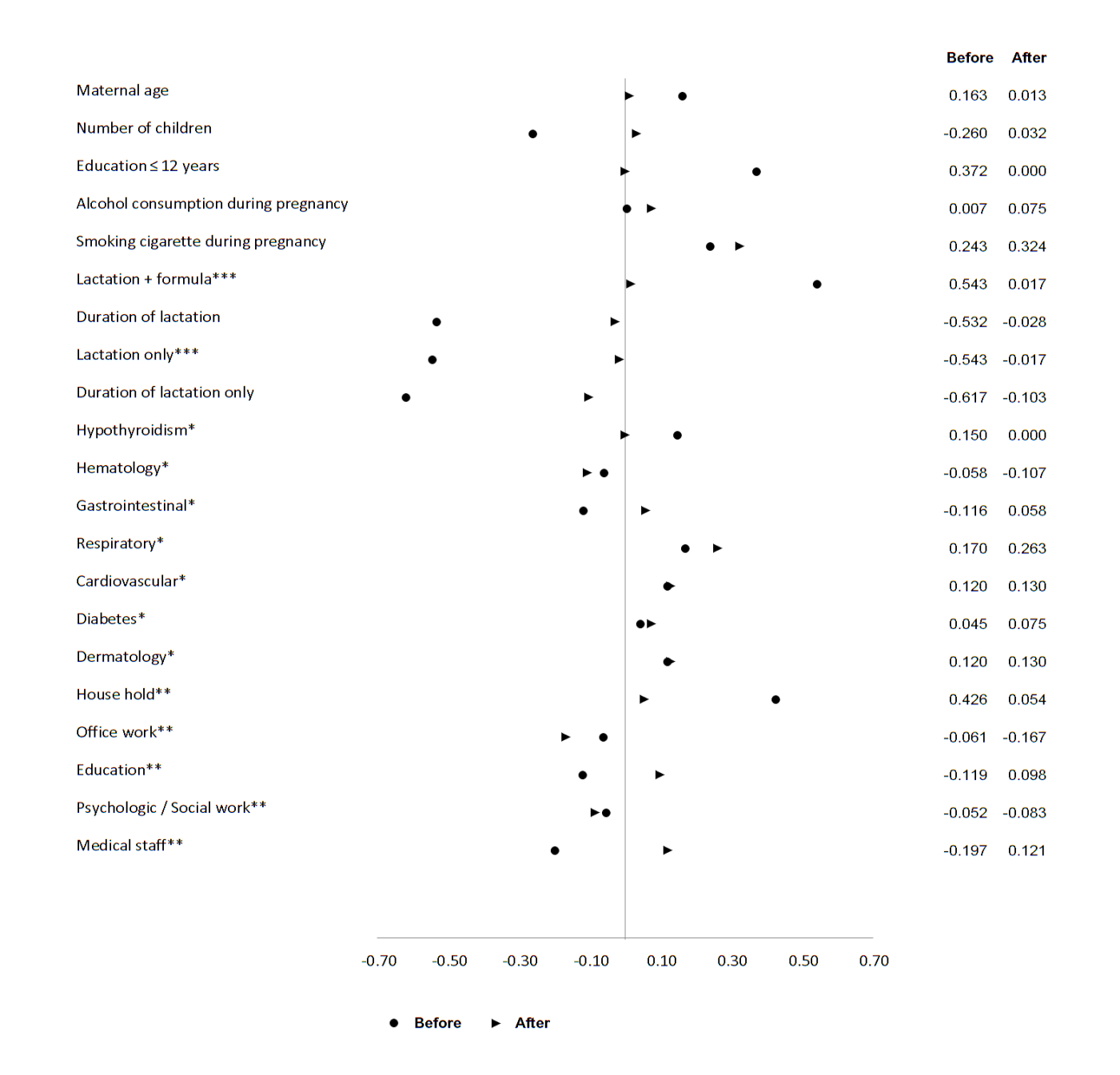
**
