# Supplementary material for: Chronic use of psychotropic medications in breastfeeding women: Is it safe?
Source: PLoS One. 2018 May 21;13(5):e0197196. doi: 10.1371/journal.pone.0197196 (PMC5962050; doi:10.1371/journal.pone.0197196)
Supplement: S3 Fig — (DOCX) [file pone.0197196.s003.docx]

**S3 Fig.** Mirrored histogram of distribution of propensity scores for Psychotropic and Antibiotic groups.


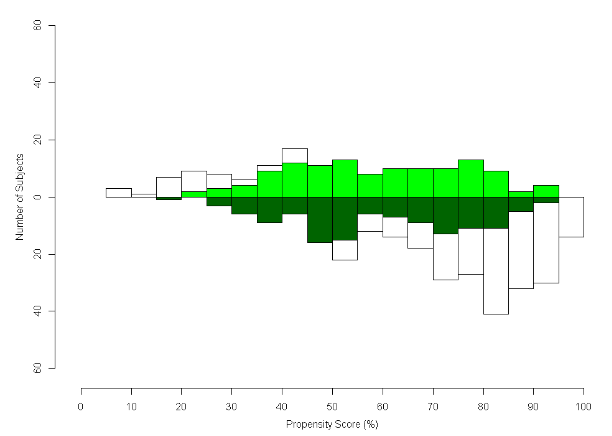


Psychotropic Group

Antibiotic Group

Psychotropic Group
