# Supplementary material for: Chronic use of psychotropic medications in breastfeeding women: Is it safe?
Source: PLoS One. 2018 May 21;13(5):e0197196. doi: 10.1371/journal.pone.0197196 (PMC5962050; doi:10.1371/journal.pone.0197196)
Supplement: S1 Table — (DOCX) [file pone.0197196.s004.docx]

|  | **Psychotropic group (N = 466)** | |  |
| --- | --- | --- | --- |
| **Characteristic** | **No response (N = 71)** | **Response (N = 395)** | **P Value** |
| Maternal age, median (IQR), y | 35 (30-38) | 35 (32-39) | 0.041 |
| Education<=12 years, No. (%) | 8 (33.3%) | 140 (35.4%) | 0.834 |
| Profession, No. (%) |  | | 0.328 |
| House hold | 3 (12.5%) | 100 (25.3%) |  |
| Office work | 11 (45.8%) | 166 (42.0%) |  |
| Education | 4 (16.7%) | 76 (19.2%) |  |
| Psychologic/Sociologic work | 1 (4.2%) | 11 (2.8%) |  |
| Medial staff | 5 (20.8%) | 42 (10.6%) |  |
| No. of pregnancies, median (IQR) | 2 (1-3) | 2 (1-3) | 0.358 |
| No. of children, median (IQR) | 1 (1-2) | 2 (1-3) | <0.001 |
| Chronic maternal illness, No. (%) |  | | |
| Cardiovascular | 0 (0%) | 4 (1%) | >0.999 |
| Hypothyroidism | 0 (0%) | 15 (3.8%) | 0.143 |
| Dermatology | 0 (0%) | 3 (0.8%) | >0.999 |
| Diabetes | 0 (0%) | 6 (1.5%) | 0.597 |
| GI | 0 (0%) | 5(1.3%) | >0.999 |
| Urinary | 0 (0%) | 0 (%) | NA |
| Hematology | 0 (0%) | 8 (2%) | 0.614 |
| Respiratory | 0 (0%) | 7 (1.8%) | 0.602 |

**S1 Tables. Baseline Characteristics of Mothers included in the Psychotropic group and Antibiotic group who responded and non-responded to the follow up interview**

Abbreviations: NA= not applicable

|  | **Antibiotic group (N = 160)** | |  |
| --- | --- | --- | --- |
| **Characteristic** | **No response (N = 8)** | **Response (N = 152)** | **P Value** |
| Maternal age , median (IQR), y | 31 (28-35) | 35 (31-38) | 0.035 |
| Chronic maternal illness, No. (%) |  | | |
| Cardiovascular | 0 (0%) | 0 (0%) | NA |
| Hypothyroidism | 0 (0%) | 3 (2%) | >0.999 |
| Dermatology | 0 (0%) | 0 (0%) | NA |
| Diabetes | 0 (0%) | 1 (0.7%) | >0.999 |
| GI | 0 (0%) | 4 (2.6%) | >0.999 |
| Urinary | 0 (0%) | 0 (0%) | NA |
| Hematology | 0 (0%) | 4 (2.6%) | >0.999 |
| Respiratory | 0 (0%) | 0 (0%) | NA |
|  |  |  |  |

Abbreviations: NA= not applicable
