# Supplementary material for: Chronic use of psychotropic medications in breastfeeding women: Is it safe?
Source: PLoS One. 2018 May 21;13(5):e0197196. doi: 10.1371/journal.pone.0197196 (PMC5962050; doi:10.1371/journal.pone.0197196)
Supplement: S2 Table — (DOCX) [file pone.0197196.s005.docx]

**S2 Table. Rate of perinatal complications in women exposed to psychotropic medications or antibiotics during childbirth**.

| **Parameter** | **Psychotropic Group (N=280)** | **Antibiotic Group (N= 152)** | **P value** |
| --- | --- | --- | --- |
| **Maternal complications** |  |  |  |
| Antibiotics treatment | 2 (0.7%) | 0 (0%) | 0.54 |
| Pre-eclampsia | 2 (0.7%) | 1 (0.6%) | >0.99 |
| Total (maternal) | 4 (1.4%) | 1 (0.6%) | 0.66 |
| **Infants' complications** |  |  |  |
| Prematurity (< 37 weeks) | 1 (0.4%) | 0 (0%) | >0.99 |
| Hypoglycemia (< 40 mg/dl) | 3 (1.1%) | 1 (0.6%) | >0.99 |
| Fetal distress^*^ | 15 (5.4%) | 0 (0%) | 0.002 |
| Total (Infants) | 19 (6.8%) | 1 (0.6%) | 0.003 |

Data is presented as the n for each category and (%)

*Defined by either bradycardia (< 100 bpm) or respiratory distress syndrome
