# Supplementary material for: Chronic use of psychotropic medications in breastfeeding women: Is it safe?
Source: PLoS One. 2018 May 21;13(5):e0197196. doi: 10.1371/journal.pone.0197196 (PMC5962050; doi:10.1371/journal.pone.0197196)
Supplement: S3 Table — (DOCX) [file pone.0197196.s006.docx]

**S3 Table. Height, weight, head circumference and weight- length ratio percentile at follow up of the breastfed infant before and after matching.**

|  | **Before matching** | | | **After matching** | | |
| --- | --- | --- | --- | --- | --- | --- |
| **Growth parameters** | **Psychotropic Group**  **(N = 280)** | **Antibiotic Group**  **(N = 152)** | **P value** | **Psychotropic Group**  **(N = 120)** | **Antibiotic Group**  **(N = 120)** | **P value** |
| Height percentile | 50 (15-50) | 50 (15-57) | 0.34 | 50 (11-50) | 50 (15-70) | 0.09 |
| Weight percentile | 50 (15-85) | 48 (15-60) | 0.22 | 50 (15-73) | 44 (15-60) | 0.94 |
| Head circumference percentile | 50 (15-50) | 50 (30-50) | 0.74 | 50 (15-50) | 50 (30-50) | 0.98 |
| Weight- length ratio | 50 (15-85) | 50 (15-85) | 0.42 | 50 (15-90) | 50 (15-85) | 0.45 |

Data are expressed as median (IQR)
