# Supplementary material for: Chronic use of psychotropic medications in breastfeeding women: Is it safe?
Source: PLoS One. 2018 May 21;13(5):e0197196. doi: 10.1371/journal.pone.0197196 (PMC5962050; doi:10.1371/journal.pone.0197196)
Supplement: S2 File — (DOC) [file pone.0197196.s008.doc]

**Methods-Supporting information:**

**Questionnaire**

***Clinical Pharmacology Unit***

***Assaf Harofeh Medical Center***

***Affiliated to the Sackler School of Medicine - Tel-Aviv University***

***Zerifin 70300, Israel • (972)-8-9779309***

**Follow-up Form**

**File number**:

**Date of follow up:**

**Verbal permission to participate in the study: Yes 🞏 No 🞏**

**General Information**

Date of call to the DCC: _______________ pregnancy/lactation ____________

Maternal Age (y): _____

Years of schooling____ Maternal profession ______________

Number pregnancies: ______ Number of children: _______

Chronic maternal illness

| Particular disease | Type of disease |
| --- | --- |
|  | Thyroid |
|  | Hypertension |
|  | Respiratory |
|  | Diabetes |
|  | Cardiovascular |
|  | Dermatology |
|  | Hematology |

**Exposures during pregnancy**

Alcohol (N/Y-freq):__________________________

Cigarettes (N/Y-freq):_________________________

Drug (N/Y-freq) : _____________________________

Drugs during pregnancy:

| Rx Medications | Indication | Dose |
| --- | --- | --- |
|  |  |  |
|  |  |  |

**Delivery Information**

Gestational age of birth: _____ Birth date: ________________

Male / female: ________ Birth weight: __________________

1. Delivery method: [ ] normal delivery

[ ] Vacuum

[ ] Caesarian- elective

[ ] Caesarian- emergency

Fetal monitoring [ ] yes [ ] no

Childbirth’s Complications

| Complication | Yes |
| --- | --- |
| Antibiotic treatment |  |
| jaundice |  |
| RDS |  |
| Premature baby |  |
| Others |  |

**Breastfeeding information**

Feeding (Breast / Bottle):______________________

Duration of breastfeeding (# weeks): ________

Duration of exclusive breastfeeding (# weeks): _______

Reason for stopping breastfeeding:

| Reasons | Indication | Dose |
| --- | --- | --- |
| Medical advice |  |  |
| No need |  |  |
| Concern |  |  |
| Milk reduction |  |  |

Medications:

| Name | Indication | Dose |
| --- | --- | --- |
|  |  |  |
|  |  |  |
|  |  |  |

Child’s Adverse reaction (ADR):

| Type of ADR | Yes |
| --- | --- |
| restlessness |  |
| shivering |  |
| seizures |  |
| somnolence |  |
| Excessive crying |  |
| diarrhea |  |
| rash |  |
| Lack of weight gain |  |
| any ADR |  |

**Gross Milestones**

(For the following give the *age* of the infant when *first*:

| Events | Age | Normal (months) |
| --- | --- | --- |
| Smiling |  | 2 |
| Lifting head |  | 3 |
| Sitting |  | 6-8 |
| Crawling |  | 8-10 |
| Standing |  | 8-10 |
| First word |  | 8-12 |
| Walking |  | 12-15 |

Infant’s age (months): _________

Infant’s length (cm): __________

Infant’s weight (kg): __________

Head circumference____
